# Impact of subclinical hypothyroidism and thyroid autoimmunity on clinical pregnancy rate after intrauterine insemination in euthyroid women

**DOI:** 10.5935/1518-0557.20190027

**Published:** 2019

**Authors:** Meryem Kuru Pekcan, A. Seval Ozgu-Erdinc, Nafiye Yilmaz

**Affiliations:** 1 University of Health Sciences, Ankara Dr. Zekai Tahir Burak Health Practice Research Center, Ankara, Turkey

**Keywords:** autoantibody, clinical pregnancy, intrauterine insemination, subclinical hypothyroidism, thyroglobulin, thyroid peroxidase

## Abstract

**Objective:**

This study aimed to evaluate the association between subclinical hypothyroidism and thyroid autoantibodies with clinical pregnancy rate after intrauterine insemination (IUI) in euthyroid women.

**Methods:**

In this prospective cohort study, we recruited 497 women who underwent IUI treatment. We assessed thyroid function tests, thyroid antibodies and clinical pregnancy rates of the patients.

**Results:**

The patients were divided into two groups according to TSH values: normal group, n=387, and subclinical hypothyroidism group 2, n=110. The clinical pregnancy rate was 15.2% in the Control Group and 17.3% in the study group (*p*=0.656). In the Study Group, 35% of the patients had anti-TPO positivity (*p*=0.531) and 42.1% of the patients had anti-TG positivity (*p*=0.285). There was no statistically significant difference in clinical pregnancy rates between the groups in terms of antithyroid antibody positivity (*p*=0.54; *p*=0.559, respectively).

**Conclusion:**

Anti-TPO antibodies and subclinical hypothyroidism had no impact on clinical pregnancy rates in the women submitted to IUI.

## INTRODUCTION

Thyroid disorders are one of the most common endocrinological diseases affecting women of reproductive age (Negro & Mestman, 2011; Krassas *et al*., 2010; Sarac & Koc, 2018). They are associated with adverse reproductive outcomes such as spontaneous abortion and infertility (Krassas *et al*., 2010; Kalem *et al*., 2016; Liu *et al*., 2005). Hypothyroidism is responsible for harmful effects on fetal health, the guidelines suggest that TSH (thyroid stimulating hormone) levels should be <2.5 mIU/L in pregnant or first trimester pregnant women (Burman, 2009; Garber *et al*., 2012). The 2012 guidelines of the American Thyroid Association and the American Society of Clinical Endocrinologists recommend limiting serum TSH to 2.5 mIU/L in euthyroid patients planning to become pregnant (Garber *et al*., 2012). However, in 2017 the American Society of Thyroid Guidelines recommended the upper reference limit of TSH to 4.0 mIU/L (Alexander *et al*., 2017). There is no clear consensus on the efficacy of an upper value for TSH and the effects on fertility outcomes (Miko *et al.,* 2017).

Chronic autoimmune thyroiditis (named Hashimoto thyroiditis) (HT), is the most common endocrinopathy in premenopausal women in developed countries (Friedrich *et al*., 2008). All over the world, iodine deficiency is still the most common cause of thyroid dysfunction (Hayashi *et al*., 1986). Various studies have shown that HT is associated with various gynecological problems, recurrent miscarriage, unexplained infertility, and *in vitro* fertilization failure (Dendrinos *et al*., 2000; Poppe *et al*., 2007; van den Boogaard *et al*., 2011; Aydın *et al*., 2008).

There is a negative association between maternal thyroid dysfunction and low birth weight, preterm birth, preeclampsia and decreased intelligence (De Groot *et al*., 2012). Subclinical hypothyroidism without thyroid autoantibodies seems to be related to the problems mentioned above (Negro *et al*., 2010; Benhadi *et al*., 2009). Therefore, hypothyroidism treatment during pregnancy is essential. Recommendations for the treatment of subclinical hypothyroidism before and during pregnancy also differ (Vila *et al*., 2014).

Intrauterine insemination (IUI) is widely used to treat infertility, and it is considered a non-invasive and less expensive treatment when compared to assisted reproduction techniques (ART) such as *in vitro* fertilization (IVF) (Dilbaz *et al.,* 2011). The clinical pregnancy rates differ between indications in 8-20% per cycle (Merviel *et al*., 2014; Dilbaz *et al*., 2011). Fertility treatment outcome in the presence of thyroid problems is challenging (Practice Committee of the American Society for Reproductive, 2015; Tan *et al*., 2014; Tuncay *et al*., 2018).

In this study, we aimed to evaluate a possible association between subclinical hypothyroidism and thyroid autoantibodies with clinical pregnancy rates after intrauterine insemination in euthyroid women.

## MATERIALS AND METHODS

### Sample and Data

We recruited 497 women who applied to the reproductive endocrinology and infertility clinics of the Zekai Tahir Burak Women's Health Education and Research Hospital from October 2015 to June 2017 in this prospective cohort study. The study was approved by the Local Ethics Committee of the institution (05.27.2015 #20), and the universal principles of the Declaration of Helsinki were applied (World Medical Association, 2013). We excluded those with tubal factor infertility, male infertility, endometriosis, and systemic disorders such as overt diabetes mellitus, cardiac pathologies and known thyroid diseases (medications such as levothyroxine or anti-thyroid drugs). Semen samples were obtained by masturbation after 2-3 days of sexual abstinence. Sperm preparation was undertaken using the swim-up technique and stored at room temperature until the time of insemination. IUI was performed using a soft IUI catheter. Semen parameters were analyzed according to the WHO 2010 criteria (Cooper *et al*., 2010). All patients received clomiphene citrate treatment (50-100 mg/day) (Klomen^@^, Kocak Farma, Istanbul, Turkey) orally or 37.5-150 IU of pure FSH or human menopausal gonadotropin (hMG) (Gonal-F^@^, Merck Sereno/ Menogon^@^, Ferring Pharmaceuticals Istanbul, Turkey), respectively starting on 3-5 cycle days of menstruation and lasting until 7-9 cycle days of menstruation for ovulation induction. The drug dosage was individualized according to patient response and/or the data from the previous cycles. We performed serial transvaginal ultrasonography (TVUS) examinations. We administered a dose of 10,000 IU of urinary HCG or 250 mg of recombinant HCG (Pregnyl^@^, Organon, Istanbul, Turkey, respectively) when at least one follicle of ≥18 mm was seen upon transvaginal ultrasonography. To prevent multiple pregnancies, we included only cycles with mono and bi-follicular growth (>18 mm) in the analysis.

We evaluated the demographic features, infertility types, infertility duration, endometrial thickness on HCG day, basal hormonal parameters (FSH, E2), thyroid function tests [free tri-iodothyronine (fT3) and free thyroxine (fT4) and TSH], thyroid antibodies [antithyroid peroxidase (anti-TPO) and antithyroglobulin (anti-TG) antibodies] and clinical pregnancy rates of the patients. We ran a qualitative serum β-HCG test 14 days after insemination if menstruation had not started. Clinical pregnancy was defined as the presence of a gestational sac with accompanying fetal heartbeat by ultrasound at least 4 weeks after IUI.

### Laboratory Analysis

Blood samples were taken from the participants' antecubital veins. All serum parameters included in this analysis were obtained on the 3rd to the 5th day of the menstrual cycle with IUI. In our department, the normal range for TSH is 0.34-5.6µIU/ml; for fT3 it is 2.5-3.9pg/ml; for fT4 it is 0.61-1.12ng/dl; 0-9IU/ml for anti-TPO; and 0-4IU/ml for anti-TG. These normal ranges were calculated by the laboratory and all examined serum parameters were determined in the ISO-certified central laboratory of the Dr. Zekai Tahir Burak Women's Health Care University of Health Sciences, Education and Research Hospital, Ankara, TURKEY, using commercially available assays using the Elecsys electrochemiluminescence immunoassays on a Cobas 6000 immunoanalyzer (Roche Diagnostics, Mannheim, Germany). The inter and intra assay CVs were <2% and <6.5% for TSH, ≤2% and <5% for fT4, ≤2% and <5% for fT3, <5% and ≤7% for anti-TPO, <2% and ≤5% for anti-TG. The TSH measuring range was 0.005-100 µIU/mL; for fT3 it was 0.3-10 nmol/L; for fT4 it was 0.101-7.77 ng/dL; for anti-TPO it was 5-600 IU/ml; for anti-TG it was 10-4000 IU/ml.

### Statistical Analysis

We used the statistical Package for the Social Sciences, version 23.0 (SPSS Inc., Chicago, IL) for the statistical analysis. The sample size calculation for the entire study population, a two-sample comparison with a 5% level of significance (alpha) and a power of 0.80 with an allocation ratio of 3:1, gave a study population of 315 *vs.* 104 women in each group. Sample size calculations were performed using the G*Power v3.1.5 general power analysis program (Faul *et al*., 2007). For quantitative data, we used mean values and standard deviations, whereas for quantitative data we used numbers and percentages. We used the Kolmogorov-Smirnov and Shapiro-Wilk tests to assess the normal distribution of the univariate variables. In order to analyze the variables that did not have normal distribution we used non-parametric methods. Non-parametric variables between groups were compared through the Mann-Whitney U test. For categorical variables we used the Fisher's exact or the Pearson Chi-Square test, where appropriate. We ran an ROC curve analysis to determine a cut-off value for pregnancy prediction. *p* values less than 0.05 indicated statistical significance. The *p-*value presented in our statistical analysis are for two-tailed tests.

## RESULTS

The area under the ROC curve revealed that no cut-off value of TSH can predict pregnancy in intrauterine insemination cycles (the area under the ROC curve was 0.495 (%95 CI: 0.424-0.566) (*p*-value=0.887). Therefore, the patients were divided into two groups as TSH values between 0.35-2.49mIU/L (group 1, n=387) and 2.52-4.88 mIU/L (group 2, n=110). The groups were statistically comparable in terms of the variables mentioned.

Demographic features of the subjects are shown in Table 1. Of these, 387 women (77.2%) had TSH values between 0.35-2.49 mIU/L (control group), and 110 women (22.8%) had TSH values between 2.52-4.88 mIU/L (study group). There were no statistically significant differences between the groups in terms of age, BMI, infertility duration, infertility type (primer/seconder), ovulation induction protocol and clinical pregnancy rates (*p*>0.05). The clinical pregnancy rate was 15.2% in the Control Group and 17.3% in the study group (*p*=0.656). There was no statistically significant difference between the groups in terms of FSH, E2 and endometrial thickness on the ovulation trigger day (Table 2).

**Table 1:** Comparison of the subjects' demographic features

	Control Group (n=387)	Study Group (n=110)	*p*-value
Age (years)	27 (18-44)	26 (19-34)	0.395**
BMI (kg/m_2_)	24.44 (16.44-40.39)	24.69 (18.73-38.71)	0.313**
Infertility duration	3 (1-14)	3 (1-14)	0.176**
Infertility			1.00*
-Primary	298 (77%)	85 (77.3%)	
-Secondary	89 (23%)	25 (22.7%)	
Ovulation Induction			0.813***
clomiphene citrate	248 (64.1%)	67 (60.9%)	
rFSH	128 (33.1%)	40 (36.4%)	
hMG	11 (2.8%)	3 (2.7%)	
Clinical pregnancy rate, n (%)	59 (15.2%)	19 (17.3%)	0.656*

* Fisher’s exact

** Mann Whitney U

**Table 2:** Laboratory parameters

	Control group (n=387)	Study group (n=110)	*p*-value
FSH	6.51 (4-15)	6.7 (3.2-55.7)	0.879**
E2	42 (5-163)	39.77 (5-84)	0.567**
Endometrial Thickness	9 (4-15)	8.5 (5-17)	0.528**
TSH	1.36 (0.35-2.49)	3.15 (2.52-4.88)	<0.0001**
fT3	3.09 (2.12-4.65)	3.16 (2.3-4.8)	0.54**
fT4	0.91 (0.56-3.24)	0.92 (0.68-1.54)	0.559**
Anti-TPO (%)	29.2%	35%	0.531*
Anti-TG (%)	29%	42.1%	0.285*

* Fisher’s exact

** Mann Whitney U

**Figure 1:**
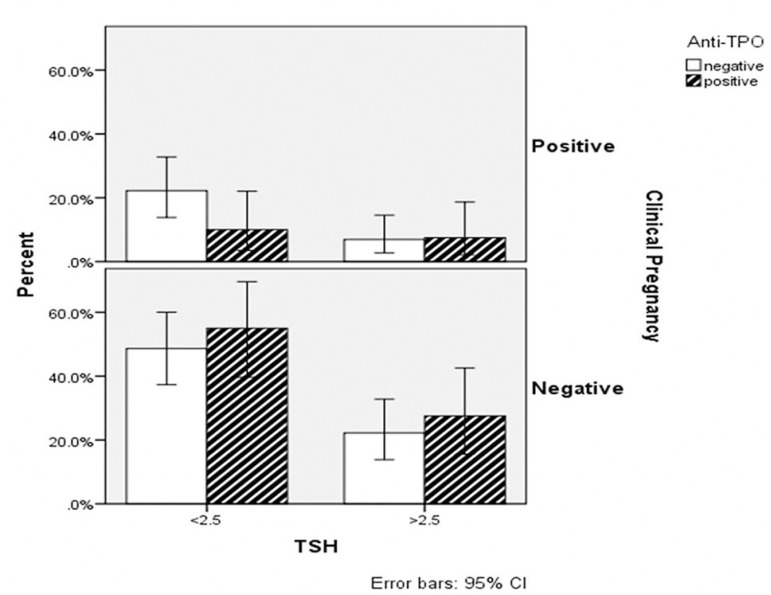
The clinical pregnancy rate of patients concerning TSH and anti-TPO positivity

**Figure 2:**
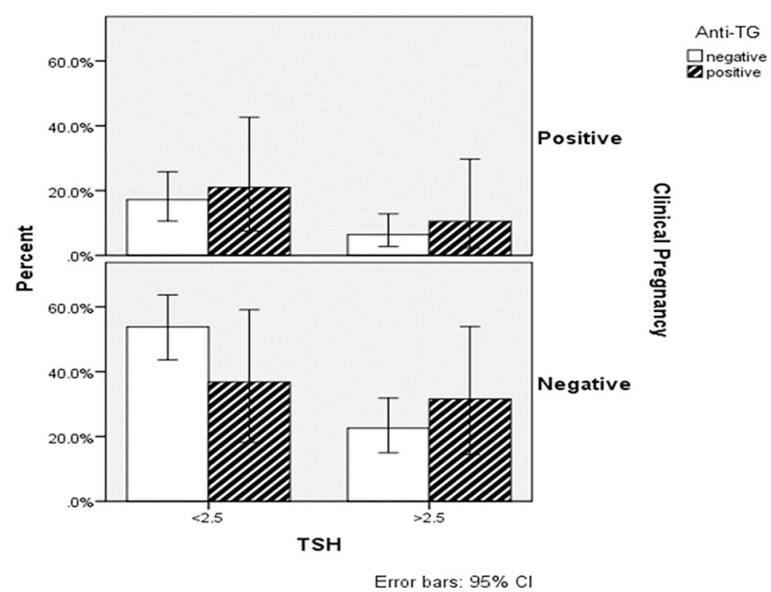
The clinical pregnancy rate of patients about TSH and anti-TG positivity

Anti-TPO positivity was present in 35% *vs.* 29.2% of patients in the Study and Control groups, respectively (*p*=0.531); while anti-TG positivity was present in 42.1% *vs.* 29% in the Study and Control groups, respectively (*p*=0.285) (Table 2). There was no statistically significant difference for clinical pregnancy rates between the groups in terms of antithyroid antibody positivity (Figures 1 and 2). No statistically significant difference between the groups was seen in terms of fT3 and fT4 results (*p*=0.54; *p*=0.559, respectively) (Table 2).

## DISCUSSION

The fertility treatment outcome in the presence of thyroid autoimmunity (TAI) and subclinical hypothyroidism is contradictory (Unuane *et al*., 2017; Medenica *et al*., 2015; Karmon *et al*., 2015; Jatzko *et al*., 2014; Tuncay *et al*., 2018). In this study, we investigated the fertility outcome in euthyroid women treated with IUI concerning the TSH threshold and antithyroid antibodies. We found no significant differences in fertility outcomes among euthyroid women between the groups. The clinical pregnancy rate was similar between the two groups. 59 patients (15.2%) out of the 397 patients in the low-TSH group (Control Group) became pregnant, whereas the clinical pregnancy rate was 19/110 (17.3%) in the subclinical hypothyroidism group (Study Group).

In the euthyroid patient group with women of normal upper TSH values we have found similar IUI outcomes compared to women with baseline TSH <2.5 mIU/L. Unexpectedly, some of the previous studies also showed results similar to those from our study; the women with a TSH score of >2.5 mIU/L before IUI had a higher birth rate after a clinical pregnancy and lower spontaneous abortion risk (Tuncay *et al*., 2018; Jatzko *et al*., 2014). Reh *et al*. (2010) observed that there was no significant difference in clinical pregnancy or birth rates between TSH levels of 0.4-2.4 mIU/L and women above 2.5 mIU/L in the infertile population. They did not report any difference in miscarriage rates in the low and high TSH groups (Reh *et al*., 2010). In another study carried out by Karmon *et al*. (2015), there was no significant difference in clinical pregnancy rates among women with TSH levels of 0.4-2.4 mIU/L and levels > 2.5 mIU/L. In addition, they found that preconceptional TSH levels were inversely associated with spontaneous abortion and positively associated with live birth after clinical pregnancy (Karmon *et al*., 2015).

The American Thyroid Association supported the 2012 guidelines on hypothyroidism management in pregnancy (Garber *et al*., 2012). The document strengthens the idea of keeping TSH levels at <2.5 mIU/L in women with hypothyroidism during the first trimester of pregnancy. Guidelines should also recommend treatment if TSH levels for euthyroid women are 2.5 mIU/L or higher in the first trimester or in those planning a pregnancy. This supports the view that physiologically HCG cross-reacts with the TSH receptor and causes a decrease in TSH levels (Gilbert *et al*., 2008).

In addition, many studies have redefined the TSH reference intervals in pregnancy and argued that there should be lower values in the first trimester (Springer *et al*., 2009; Garber *et al*., 2012; Ödöl et al., 2009). However, there is no evidence that pre-pregnancy outcome in early euthyroid women with high normal TSH levels has altered early cycle and pregnancy outcomes. Furthermore, since general screening is not recommended, it is difficult to make a decision to intervene in the high normal TSH values found incidentally in a non-pregnant asymptomatic patient (Committee on Patient Safety and Quality Improvement; Committee on Professional Liability, 2007).

Recent studies in pregnant women in Asia (China, Korea, and India) have shown that there is only a minimal reduction in the upper reference level (Li *et al*., 2014; Moon *et al*., 2015). According to these results, in the recent guidelines of the American Thyroid Association, the lower reference range of TSH decreased by about 0.4 mIU/L, the upper reference range decreased by about 0.5 mIU/L. This corresponds to a TSH upper limit of 4.0 mIU/L for patients in the first trimester (Alexander *et al*., 2017). In our study, no cut-off limit for TSH can be found to predict pregnancy. A recent guide from the Practice Committee of the American Society for Reproductive Medicine (2015) states that there is insufficient data to indicate that TSH levels between 2.5 and 4 mIU/L are associated with abortion and pregnancy side effects.

In a study by Negro *et al*. (2010), in 4,123 thyroid antibody-negative women, it was reported that the loss of pregnancy below 11 weeks was higher in people with TSH levels of 2.5-5 mIU/L. There may be a few reasons for this. The authors did not work on the infertile population, but included women who were in their first trimester with spontaneous pregnancies. In their study, all TSH levels were measured in the preconceptional period. This difference can be explained in part as follows; women with TSH levels ≥2.5 mIU/L in the first trimester may have higher levels before pregnancy if TSH drops in early gestation, as suggested in the literature (Gilbert *et al*., 2008). In addition, over-stimulation appears to influence TSH levels (Gracia *et al*., 2012). Future studies should clarify the potential benefits of treatment of women with high normal TSH levels who are already pregnant and asymptomatic, or who plan to become pregnant (naturally or otherwise).

The strengths of this study include the large sample size and its unique population of women undergoing IUI, which allowed the uniform assessment of preconceptional levels of TSH. A related point is that all the patients in our center routinely undergo TSH measurement before receiving IUI treatments.

Despite these advantages, an associated limitation is that the live birth, spontaneous abortion and other obstetric or fetal end points of our subjects were not available. Further evaluation of this relation is necessary to rule out the possibility of chance and unmeasured confounding.

TSH and TAI were independently associated with pregnancy outcomes after spontaneous conception or ART (Thangaratinam *et al*., 2011). One review showed antithyroid antibodies were not associated with increased reproductive loss in patients submitted to ART treatments (Leiva *et al*., 2017). In two meta-analyses carried out with ART (Busnelli *et al*., 2016; Toulis *et al*., 2010), TAI has a potentially harmful effect on pregnancy. In a meta-analysis involving twelve studies, Busnelli *et al*. (2016) showed a negative TAI effect of in terms of an increased risk of miscarriage and a decreased chance of live birth. In another meta-analysis involving four studies, Toulis *et al*. (2010) showed a 2-fold increase in risk of miscarriage for TAI-positive patients, but no significant effect on clinical pregnancy and live birth rates. In another study, pregnancy outcomes of 114 TAI-positive and 495 TAI-negative infertile women were compared and there was no significant difference in implantation, fertilization rate, pregnancy rates and live birth rates (Łukaszuk et al., 2015). Tan *et al*. (2014) concluded that pregnancy outcome was comparable between women with and without TAI after-ICSI, but TAI status did not affect ICSI outcomes alone.

Several hypotheses have been proposed to explain the possible causal relationship between TAI and negative obstetric outcome. First, TAI can lead to a general immune imbalance, implantation failure targeting the reproductive tract. Thus, thyroid antibodies are considered to be among the causes of fertility problems and recurrent pregnancy loss. Second, thyroid antibodies may cause thyroid function decline as an undesirable pregnancy outcome. A positive TAI status increases the risk of developing (sub) clinical hypothyroidism (Medici *et al*., 2014). In Unuane's study, the baseline characteristics of both patient groups were similar. There was a significant higher mean TSH in the anti-TPO positive group upon the fertility treatment onset (Unuane *et al*., 2017).

In conclusion, with this large prospective cohort study we could not find any significant difference in clinical pregnancy rates in women with and without anti-TPO antibodies and subclinical hypothyroidism who underwent IUI. We could not confirm that a TSH level above 2.5 mIU/l has a negative effect on pregnancy rates. More prospective studies are needed to confirm our results, which will shed new light on the impact of thyroid function on IUI success. Future studies will also be useful to clarify which TSH threshold for thyroid hormone replacement should be used for infertile women.

## References

[r1] Alexander EK, Pearce EN, Brent GA, Brown RS, Chen H, Dosiou C, Grobman WA, Laurberg P, Lazarus JH, Mandel SJ, Peeters RP, Sullivan S (2017). 2017 Guidelines of the American Thyroid Association for the
Diagnosis and Management of Thyroid Disease During Pregnancy and the
Postpartum. Thyroid.

[r2] Aydın A, Dönmez M, Aydın Y, Karatekin G, Özdemir G, Oruç Ö (2008). Thyroid Storm Complicating Pregnancy, A Case Report and
Management. Gynecol Obstet Reprod Med.

[r3] Benhadi N, Wiersinga WM, Reitsma JB, Vrijkotte TG, Bonsel GJ (2009). Higher maternal TSH levels in pregnancy are associated with
increased risk for miscarriage, fetal or neonatal death. Eur J Endocrinol.

[r4] Burman KD (2009). Controversies surrounding pregnancy, maternal thyroid status, and
fetal outcome. Thyroid.

[r5] Busnelli A, Paffoni A, Fedele L, Somigliana E (2016). The impact of thyroid autoimmunity on IVF/ICSI outcome: a
systematic review and meta-analysis. Hum Reprod Update.

[r6] Committee on Patient Safety and Quality Improvement, Committee on Professional Liability (2007). ACOG Committee Opinion No. 381: Subclinical hypothyroidism in
pregnancy. Obstet Gynecol.

[r7] Cooper TG, Noonan E, von Eckardstein S, Auger J, Baker HW, Behre HM, Haugen TB, Kruger T, Wang C, Mbizvo MT, Vogelsong KM (2010). World Health Organization reference values for human semen
characteristics. Hum Reprod Update.

[r8] De Groot L, Abalovich M, Alexander EK, Amino N, Barbour L, Cobin RH, Eastman CJ, Lazarus JH, Luton D, Mandel SJ, Mestman J, Rovet J, Sullivan S (2012). Management of thyroid dysfunction during pregnancy and
postpartum: an Endocrine Society clinical practice guideline. J Clin Endocrinol Metab.

[r9] Dendrinos S, Papasteriades C, Tarassi K, Christodoulakos G, Prasinos G, Creatsas G (2000). Thyroid autoimmunity in patients with recurrent spontaneous
miscarriages. Gynecol Endocrinol.

[r10] Dilbaz B, Özkaya E, Çınar M, Çakır E, Dilbaz S (2011). Predictors of Total Gonadotropin Dose Required for Follicular
Growth in Controlled Ovarian Stimulation with Intrauterin Insemination
Cycles in Patients with Unexplained Infertility or Male
Subfertility. Gynecol Obstet Reprod Med.

[r11] Faul F, Erdfelder E, Lang AG, Buchner A (2007). G*Power 3: a flexible statistical power analysis program for the
social, behavioral, and biomedical sciences. Behav Res Methods.

[r12] Friedrich N, Schwarz S, Thonack J, John U, Wallaschofski H, Völzke H (2008). Association between parity and autoimmune thyroiditis in a
general female population. Autoimmunity.

[r13] Garber JR, Cobin RH, Gharib H, Hennessey JV, Klein I, Mechanick JI, Pessah-Pollack R, Singer PA, Woeber KA, American Association of Clinical Endocrinologists, American Thyroid Association Taskforce on Hypothyroidism in
Adults (2012). Clinical practice guidelines for hypothyroidism in adults:
cosponsored by the American Association of Clinical Endocrinologists and the
American Thyroid Association. Endocr Pract.

[r14] Gilbert RM, Hadlow NC, Walsh JP, Fletcher SJ, Brown SJ, Stuckey BG, Lim EM (2008). Assessment of thyroid function during pregnancy: first-trimester
(weeks 9-13) reference intervals derived from Western Australian
women. Med J Aust.

[r15] Gracia CR, Morse CB, Chan G, Schilling S, Prewitt M, Sammel MD, Mandel SJ (2012). Thyroid function during controlled ovarian hyperstimulation as
part of in vitro fertilization. Fertil Steril.

[r16] Hayashi N, Tamaki N, Konishi J, Yonekura Y, Senda M, Kasagi K, Yamamoto K, Iida Y, Misaki T, Endo K, Torizuka K, Mori T (1986). Sonography of Hashimoto's thyroiditis. J Clin Ultrasound.

[r17] Jatzko B, Vytiska-Bistorfer E, Pawlik A, Promberger R, Mayerhofer K, Ott J (2014). The impact of thyroid function on intrauterine insemination
outcome--a retrospective analysis. Reprod Biol Endocrinol.

[r18] Kalem MN, Kalem Z, Gürgan T (2016). Problems and Complications During the Treatment of Infertility in
Women with Polycystic Ovary Syndrome. Gynecol Obstet Reprod Med.

[r19] Karmon AE, Batsis M, Chavarro JE, Souter I (2015). Preconceptional thyroid-stimulating hormone levels and outcomes
of intrauterine insemination among euthyroid infertile women. Fertil Steril.

[r20] Krassas GE, Poppe K, Glinoer D (2010). Thyroid function and human reproductive health. Endocr Rev.

[r21] Leiva P, Schwarze JE, Vasquez P, Ortega C, Villa S, Crosby J, Balmaceda J, Pommer R (2017). There is no association between the presence of anti-thyroid
antibodies and increased reproductive loss in pregnant women after ART: a
systematic review and meta-analysis. JBRA Assist Reprod.

[r22] Li C, Shan Z, Mao J, Wang W, Xie X, Zhou W, Li C, Xu B, Bi L, Meng T, Du J, Zhang S, Gao Z, Zhang X, Yang L, Fan C, Teng W (2014). Assessment of thyroid function during first-trimester pregnancy:
what is the rational upper limit of serum TSH during the first trimester in
Chinese pregnant women?. J Clin Endocrinol Metab.

[r23] Liu J, Larsen U, Wyshak G (2005). Prevalence of primary infertility in China: in-depth analysis of
infertility differentials in three minority province/autonomous
regions. J Biosoc Sci.

[r24] Łukaszuk K, Kunicki M, Kulwikowska P, Liss J, Pastuszek E, Jaszczołt M, Męczekalski B, Skowroński K (2015). The impact of the presence of antithyroid antibodies on pregnancy
outcome following intracytoplasmatic sperm injection-ICSI and embryo
transfer in women with normal thyreotropine levels. J Endocrinol Invest.

[r25] Medenica S, Nedeljkovic O, Radojevic N, Stojkovic M, Trbojevic B, Pajovic B (2015). Thyroid dysfunction and thyroid autoimmunity in euthyroid women
in achieving fertility. Eur Rev Med Pharmacol Sci.

[r26] Medici M, Korevaar TI, Schalekamp-Timmermans S, Gaillard R, de Rijke YB, Visser WE, Visser W, de Muinck Keizer-Schrama SM, Hofman A, Hooijkaas H, Bongers-Schokking JJ, Tiemeier H, Jaddoe VW, Visser TJ, Peeters RP, Steegers EA (2014). Maternal early-pregnancy thyroid function is associated with
subsequent hypertensive disorders of pregnancy: the generation R
study. J Clin Endocrinol Metab.

[r27] Merviel P, Cabry R, Lourdel E, Barbier F, Scheffler F, Mansouri N, Devaux A, Benkhalifa M, Copin H (2014). Intrauterine insemination. Rev Prat.

[r28] Miko E, Meggyes M, Doba K, Farkas N, Bogar B, Barakonyi A, Szereday L, Szekeres-Bartho J, Mezosi E (2017). Characteristics of peripheral blood NK and NKT-like cells in
euthyroid and subclinical hypothyroid women with thyroid autoimmunity
experiencing reproductive failure. J Reprod Immunol.

[r29] Moon HW, Chung HJ, Park CM, Hur M, Yun YM (2015). Establishment of trimester-specific reference intervals for
thyroid hormones in Korean pregnant women. Ann Lab Med.

[r30] Negro R, Schwartz A, Gismondi R, Tinelli A, Mangieri T, Stagnaro-Green A (2010). Increased pregnancy loss rate in thyroid antibody negative women
with TSH levels between 2.5 and 5.0 in the first trimester of
pregnancy. J Clin Endocrinol Metab.

[r31] Negro R, Mestman JH (2011). Thyroid disease in pregnancy. Best Pract Res Clin Endocrinol Metab.

[r32] Ödöl E, Tosun M, Torumtay B, Alper T, Malatyalıoğlu E, Çetinkaya M, Kökçü A (2009). Antenatal Screening for the Frequency of Subclinic
Hypothroidism. Gynecol Obstet Reprod Med.

[r33] Poppe K, Velkeniers B, Glinoer D (2007). Thyroid disease and female reproduction. Clin Endocrinol (Oxf).

[r34] Practice Committee of The American Society For Reproductive
Medicine (2015). Subclinical hypothyroidism in the infertile female population: a
guideline. Fertil Steril.

[r35] Reh A, Grifo J, Danoff A (2010). What is a normal thyroid-stimulating hormone (TSH) level? Effects
of stricter TSH thresholds on pregnancy outcomes after in vitro
fertilization. Fertil Steril.

[r36] Sarac M, Koc I (2018). Prevalence and Risk Factors of Infertility in Turkey: Evidence
from Demographic and Health Surveys, 1993-2013. J Biosoc Sci.

[r37] Springer D, Zima T, Limanova Z (2009). Reference intervals in evaluation of maternal thyroid function
during the first trimester of pregnancy. Eur J Endocrinol..

[r38] Tan S, Dieterle S, Pechlavanis S, Janssen OE, Fuhrer D (2014). Thyroid autoantibodies per se do not impair intracytoplasmic
sperm injection outcome in euthyroid healthy women. Eur J Endocrinol.

[r39] Thangaratinam S, Tan A, Knox E, Kilby MD, Franklyn J, Coomarasamy A (2011). Association between thyroid autoantibodies and miscarriage and
preterm birth: meta-analysis of evidence. BMJ.

[r40] Toulis KA, Goulis DG, Venetis CA, Kolibianakis EM, Negro R, Tarlatzis BC, Papadimas I (2010). Risk of spontaneous miscarriage in euthyroid women with thyroid
autoimmunity undergoing IVF: a meta-analysis. Eur J Endocrinol.

[r41] Tuncay G, Karaer A, İnci Coşkun E, Baloğlu D, Tecellioğlu AN (2018). The impact of thyroid-stimulating hormone levels in euthyroid
women on intrauterine insemination outcome. BMC Womens Health.

[r42] Unuane D, Velkeniers B, Bravenboer B, Drakopoulos P, Tournaye H, Parra J, De Brucker M (2017). Impact of thyroid autoimmunity in euthyroid women on live birth
rate after IUI. Hum Reprod.

[r43] van den Boogaard E, Vissenberg R, Land JA, van Wely M, van der Post JA, Goddijn M, Bisschop PH (2011). Significance of (sub)clinical thyroid dysfunction and thyroid
autoimmunity before conception and in early pregnancy: a systematic
review. Hum Reprod Update.

[r44] Vila L, Velasco I, González S, Morales F, Sánchez E, Torrejón S, Soldevila B, Stagnaro-Green A, Puig-Domingo M (2014). Controversies in endocrinology: On the need for universal thyroid
screening in pregnant women. Eur J Endocrinol.

[r45] World Medical Association (2013). World Medical Association Declaration of Helsinki: ethical
principles for medical research involving human subjects. JAMA.

